# Effect of the spider toxin Tx3-3 on spinal processing of sensory information in naive and neuropathic rats: an in vivo electrophysiological study

**DOI:** 10.1097/PR9.0000000000000610

**Published:** 2017-07-06

**Authors:** Gerusa D. Dalmolin, Kirsty Bannister, Leonor Gonçalves, Shafaq Sikandar, Ryan Patel, Marta do Nascimento Cordeiro, Marcus Vinícius Gomez, Juliano Ferreira, Anthony H. Dickenson

**Affiliations:** a Department of Neuroscience, Physiology and Pharmacology, University College London, London, United Kingdom. Dalmolin is now with Department of Pharmacology, Federal University of Santa Catarina, Florianópolis, SC, Brazil. Sikandar is now with Wolfson Institute of Biomedical Research, University College London, London, United Kingdom; b Ezequiel Dias Foundation, Belo Horizonte, MG, Brazil; c Research Institute of Santa Casa de Belo Horizonte, Belo Horizonte, MG, Brazil; d Department of Pharmacology, Federal University of Santa Catarina, Florianópolis, SC, Brazil

**Keywords:** Voltage-gated calcium channel, Neuropathic pain, Spinal cord, Peptide toxin, In vivo electrophysiology

## Abstract

**Introduction:**

Drugs that counteract nociceptive transmission in the spinal dorsal horn preferentially after nerve injury are being pursued as possible neuropathic pain treatments. In a previous behavioural study, the peptide toxin Tx3-3, which blocks P/Q- and R-type voltage-gated calcium channels, was effective in neuropathic pain models.

**Objectives:**

In the present study, we aimed to investigate the effect of Tx3-3 on dorsal horn neuronal responses in rats under physiological conditions and neuropathic pain condition induced by spinal nerve ligation (SNL).

**Methods:**

In vivo electrophysiological recordings of dorsal horn neuronal response to electrical and natural (mechanical and thermal) stimuli were made in rats under normal physiological state (naive rats) or after the SNL model of neuropathic pain.

**Results:**

Tx3-3 (0.3–100 pmol/site) exhibited greater inhibitory effect on electrical-evoked neuronal response of SNL rats than naive rats, inhibiting nociceptive C-fibre and Aδ-fibre responses only in SNL rats. The wind-up of neurones, a measurement of spinal cord hyperexcitability, was also more susceptible to a dose-related inhibition by Tx3-3 after nerve injury. Moreover, Tx3-3 exhibited higher potency to inhibit mechanical- and thermal-evoked neuronal response in conditions of neuropathy.

**Conclusion:**

Tx3-3 mediated differential inhibitory effect under physiological and neuropathic conditions, exhibiting greater potency in conditions of neuropathic pain.

## Abstract 2

The P/Q- and R-type voltage-gated calcium channel blocker Tx3-3 inhibits dorsal horn neuronal response of rats with greater potency after nerve injury.

## 1. Introduction

Natural products have been historically employed in the alleviation of pain. In recent years, the identification of drugs with analgesic properties from animal venoms has resulted in novel structural classes and mechanisms of actions. Voltage-gated calcium channels (VGCCs) emerged as potential targets to treat severe pain conditions with the advent of a peptide toxin extracted from the venom of the cone snail *Conus magus*, the ω-conotoxin MVIIA, as a treatment for severe pain refractory to other treatments.^31,44,46^ The ω-conotoxin MVIIA (synthetic form known as ziconotide, Prial) produces pain relief by blocking N-type VGCC.^35^

Voltage-gated calcium channels comprise a family of ion channels divided into low-threshold (T-type) and high-threshold (L-, N-, P/Q-, and R-type) voltage-gated channels. Based on the sequence homology of the main pore-formed α1 subunit, VGCCs are divided into 3 families: Cav1, Cav2, and Cav3.^16^ The Cav2 family includes VGCCs that carry P/Q- (Cav2.1), N- (Cav2.2), and R-type (Cav2.3) currents. These currents are distinguished by their sensitivity to peptide toxins from snail and spider venoms^18,36,41^ and are present primarily in neurones, being involved in neurotransmitter release and synaptic plasticity.^7,8^ In the spinal cord, VGCCs are distributed among various types of neurones and have been implicated in the spinal processing of nociceptive transmission.^57^

Different subtypes of VGCC blockers have been tested in animal models of pain, and the responsiveness of each subtype of VGCC blockers often depends on the nature of the pain state.^10,28,45,50,54^ Notably, under neuropathic pain conditions, spinal N- and R-type calcium currents seem to play an important role.^29,30^ On the other hand, spinal P/Q-type VGCCs are less implicated in dorsal horn neuronal response after nerve damage^30^ but are related to central sensitization secondary to inflammatory pain.^32,33^ In line with electrophysiological studies, knockout animals have highlighted the role of the different VGCC subtypes in transmission and sensitization of pain.^27,42,43^

Recently, the peptide toxin Tx3-3, extracted from the venom of the spider *Phoneutria nigriventer* was tested in a behavioural study in rodents and showed antinociceptive effect in experimental neuropathic pain rather than inflammatory pain.^13^ Tx3-3 blocks P/Q- and R-type VGCCs^24^ but induces a different profile of behavioural effects when compared to the P/Q-blocker ω-conotoxin MVIIC, exhibiting better therapeutic window. Although ω-conotoxin MVIIC elicited serious motor dysfunctions when injected by intrathecal (i.t.) or intracerebroventricular (i.c.v) routes, injection of Tx3-3 induced minimal motor effects by i.c.v route, only at doses of an order of magnitude higher than the antinociceptive dose.^13^ The therapeutic window exhibited by i.t. Tx3-3 in previous behavioural experiments (no side effects were elicited with a dose >10 times the median effective dose)^13^ is interesting in the search of clinically useful agents, given that most of VGCC blockers studied have a narrow therapeutic window.^28,39^ However, whether this peptide toxin directly affects nociceptive transmission in dorsal horn spinal cord is not known. Furthermore, drugs that counteract the nociceptive transmission in the spinal dorsal horn preferentially after nerve injury are being pursued as possible neuropathic pain treatments. Therefore, the aim of the present study was to investigate the effects of Tx3-3 on the evoked dorsal horn neuronal responses in normal physiological conditions and in neuropathic conditions induced by spinal nerve ligation (SNL) in rats.

## 2. Materials and methods

### 2.1. Animals

Thirty nine male Sprague Dawley rats (Central Biologic Services, University College London, London, United Kingdom), weighing 200–250 g, were used in this study. They were housed at a maximum of 5 per cage on a 12-hour day/night cycle. Food and water were available *ad libitum*. All experimental procedures were approved by the UK Home Office and follow the guidelines of the International Association for the Study of Pain for the care and use of laboratory animals.^59^ The number of animals used was the minimum necessary to demonstrate consistent effects of drug treatments.

### 2.2. Toxin purification

*Phoneutria nigriventer* venom was obtained by electrical stimulation of anesthetized spiders. The peptide toxin Tx3-3 was purified from the venom by a combination of chromatographic steps, as described previously.^12^ The criteria for the purity and identity of the Tx3-3 are based on the shape/morphology of the toxin peak in the final reverse phase high pressure liquid chromatography (RP-HPLC), the results of electrospray ionization quadrupole time-of-fly mass spectrometry (ESI/TOF MS) mass spectroscopy, and the determination of the N-terminal sequence of 20–20 amino acids of every sample. All of the samples used presented a purity of better than 95%. Tx3-3 has a molecular weight of 5100.00 Da and its aminoacid sequence is GKCADAWESCDNYPCCVVNGYSRTCMCSANRCNCDDTKTLREHFG.^6^ Tx3-3 was stored at −20°C and freshly diluted to experimental doses with saline solution (NaCl 0.9%).

### 2.3. Spinal nerve ligation

Selective tight ligation of spinal nerves L5 and L6 was performed as first described by Kim and Chung.^23^ Briefly, under gaseous anaesthesia (isoflurane 1.5%–1.7%, delivered in a gaseous mix of 66% of N_2_O and 33% of O_2_), the rat was placed in a prone position and a midline incision was made from L4–S2. The left paraspinal muscles were separated from the spinous processes. Part of the L6 transverse process was removed to expose the L4 and L5 spinal nerves, and L6 was identified lying just under the sacrum. The left L5 and L6 spinal nerves were isolated using glass nerve hooks (Ski-ry Ltd, London, United Kingdom) and tightly ligated, using 6-0 silk thread, distal to their dorsal root ganglion and proximal to their conjunction to form the sciatic nerve. Haemostasis was confirmed, the wound was sutured, and the animal recovered from anaesthesia. For 2 weeks after surgery, the rats were housed in groups of 4 in plastic cages under a 12/12-hour day/night cycle, and their general health was monitored. The electrophysiological experiments were made at postoperative days 14 to 17, when neuropathic pain behaviours have been shown to be evident.^11,19,30^

### 2.4. In vivo electrophysiology

In vivo electrophysiology recordings were performed as previously described.^11^ Briefly, anaesthesia was induced (isoflurane 5%; 66% N_2_O, and 33% O_2_) and a cannula was inserted into the trachea, to allow continuous anesthetic delivery along the experiment (isoflurane 1.5%; 66% N_2_O, and 33% O_2_). This level of anaesthesia was maintained throughout experiments that lasted up to 9 hours. During this time the core body temperature of the rat was monitored and maintained (36·5–37°C) by means of a heating blanket connected to a rectal thermal probe via an automatic feedback control unit. The rats breathed spontaneously throughout the experiment and therefore were able to regulate their acid–base balance. A laminectomy was performed (vertebrae L1–L3) to expose segments L4-L5 of the spinal cord. Extracellular recordings of single deep wide dynamic range dorsal horn neurones (500–1000 μm depth; laminae V–VI) were made using a parylene-coated tungsten electrode (125-μm diameter, 2 MΩ; A-M systems). Positive wide dynamic range neurones were identified by their ability to respond to a range of von Frey filaments and thermal stimuli applied to the receptive field. Data were captured and analysed by a CED 1401 interface coupled to Spike 2 software (Cambridge Electronic Design, Cambridge, United Kingdom).

### 2.5. Experimental design

The testing protocol consisted of an electrical test followed by the natural stimuli. Electrical stimulation consisted of a train of 16 transcutaneous electrical stimuli (2 milliseconds pulse width, 0.5 Hz) applied to the receptive field at 3 times the threshold current for C-fibre activation, which generated a post-stimulus histogram in which responses evoked by Aβ-fibre (0–20 milliseconds), Aδ-fibre (20–90 milliseconds), C-fibre (90–300 milliseconds), and postdischarge (300–800 milliseconds) were separated and quantified on the basis of latency. The number of action potentials in the C-fibre and postdischarge range evoked by the first stimulus of the train multiplied by the total number of electrical stimuli (16) is referred to as input (non potentiated response). Wind-up (potentiated response) was calculated as the total number of action potentials in the C-fibre and postdischarge range produced by the train of 16 stimuli minus the input.

Following electrical stimuli, natural stimuli, comprising dynamic brush, mechanical punctate (application of von Frey filaments: 8, 15 and 60 g), and thermal stimuli (application of a constant water jet at 40, 45, and 48°C), were applied to the neuronal receptive field for 10 seconds, and the total number of evoked spikes was recorded. Sufficient intervals (1–2 minutes) were allowed within and between stimuli to avoid sensitization of receptors. After 3 consecutive stable control trials (<20% variation for all parameters), neuronal responses were averaged to give predrug control values with which subsequent responses were compared. Spinal applications of Tx3-3 (0.03–100 pmol/site, in a volume of 50 μL) were made directly onto the exposed surface of the spinal cord. The doses of Tx3-3 used were chosen based on previous behavioural study.^13^ The effect of each dose was followed for an hour, with tests carried out at 10, 30, and 60 minutes post-drug. One neurone per rat was recorded in each experiment. In SNL rats, all neurones recorded had the receptive field over the left hindpaw, ipsilateral to surgery. In naive rats, either left or right side of spinal cord was analysed, in a counterbalanced manner.

### 2.6. Statistical analysis

Statistical analysis was performed using GraphPad Prism Version 5.01. One-way analysis of variance (ANOVA) with Dunnett's post hoc test was used in dose–response curve experiments; 2-way ANOVA was used to access the effect of Tx3-3 on wind-up response; Student's paired *t* test was used to access the effect of Tx3-3 on input responses; Student's unpaired *t* test was used to compare neuronal characteristics and evoked responses between naive and SNL rats. F and *P* values are given at maximum peak of each significant interval; *P* < 0.05 were considered significant.

## 3. Results

The effects of Tx3-3 applied directly onto the spinal cord on the electrically, mechanically and thermally evoked response of deep dorsal horn neurones were tested in naive and SNL rats. No significant difference was found between the 2 experimental groups in the mean cell depth of recorded neurones and the mean baseline neuronal responses evoked by electrical, mechanical, and noxious thermal stimulation (Table 1), allowing direct comparison of the effects of Tx3-3 between the 2 experimental groups.

**Table 1:**
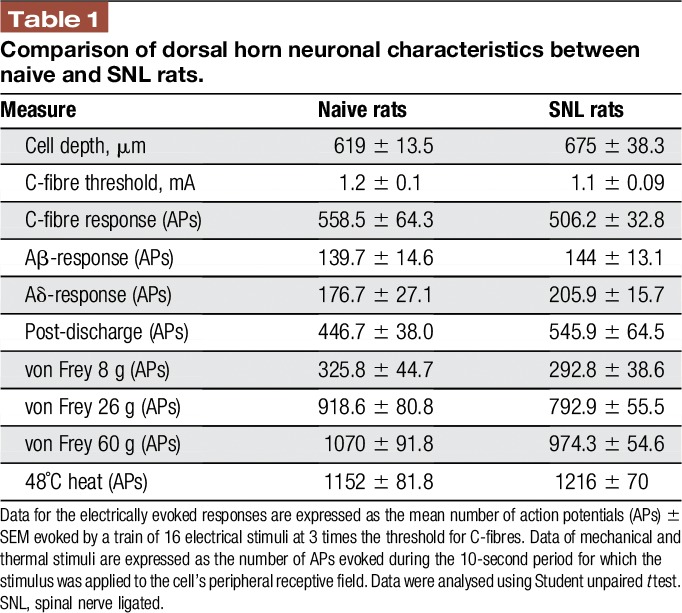
Comparison of dorsal horn neuronal characteristics between naive and SNL rats.

### 3.1. Tx3-3 shows greater inhibition profile on electrically evoked neuronal responses in spinal nerve ligated than naive rats

**Figure 1.**
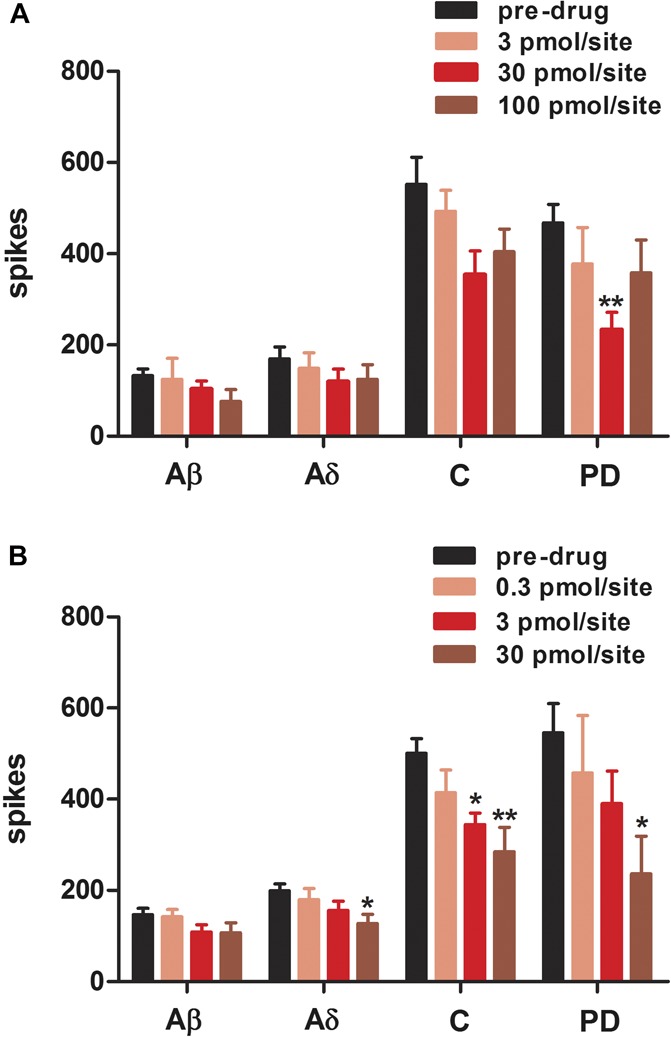
Dose–response curve of Tx3-3 on the electrically evoked neuronal responses recorded from naive (A) and SNL (B) rats. Aβ-fibre (Aβ), Aδ-fibre (Aδ), C-fibre (C) and postdischarge (PD) measurements are shown. Data are expressed as mean maximum inhibition response during the first hour after drug application onto spinal cord of rats (50 µL/site) ± SEM. In spinal nerve ligation rats, the neuronal response was evaluated 14 to 17 days after surgery. Each column represents the mean response of 5 to 7 neurones. Vertical lines show the SEM. Statistical analysis was determined using 1-way analysis of variance followed by Dunnett's post hoc test. Asterisks denote the significance levels in comparison to predrug value, *P < 0.05, **P < 0.01.

**Figure 2.**
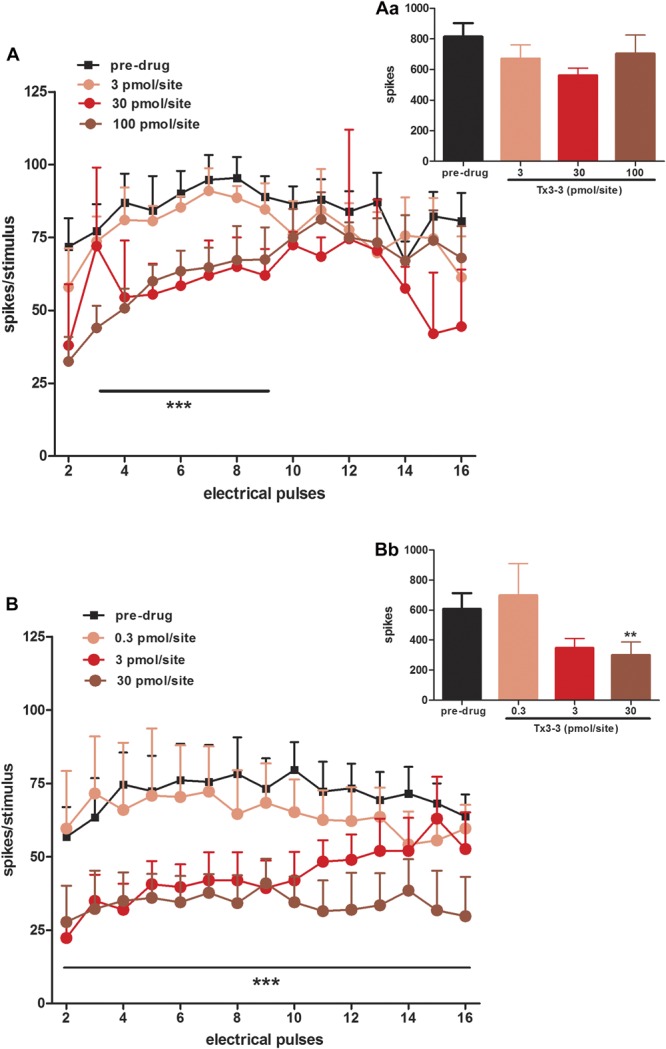
Effect of spinally applied Tx3-3 on the electrically evoked neuronal wind-up and input responses recorded from naive (A, Aa) and spinal nerve ligation (B, Bb) rats. Data are expressed as mean number of spikes evoked per stimulus of the electrical train of 16 stimuli (wind-up; A, B) and mean number of spikes evoked by the first stimulus of the electrical train × 16 (input; Aa, Bb) ± SEM. Each point or column represents the mean response of 5 to 7 neurones. Vertical lines show the SEM. Statistical analysis was determined using 2-way analysis of variance for wind-up response (A, B) and paired *t* test for input response (Aa, Bb). Asterisks denote the significance levels in comparison to predrug values, **P < 0.01, ***P < 0.001.

Tx3-3 caused an inhibitory effect on the dorsal horn neuronal responses to electrical stimulation in SNL rats and, to a far lesser extent, in naive animals. In naive rats, Tx3-3 (30 pmol/site) mediated statistically significant inhibitions of postdischarge response, compared to predrug values (45 ± 10% inhibition; *F*_(3,25)_ = 3.704, *P* = 0.0248), but no effect was seen on Aβ, Aδ, and C-fibre responses (Fig. 1A). In SNL rats, Tx3-3 caused a clear dose-dependent inhibition of postdischarge (50.6 ± 16.08% inhibition; *F*_(3,25)_ = 3.081, *P* = 0.0457), C-fibre (37.3 ± 13.97% inhibition; *F*_(3,25)_ = 6.286, *P* = 0.0025), and Aδ- (27.9 ± 8.1% inhibition; *F*_(3,25)_ = 3.011, *P* = 0.049) response, but no effect was seen on Aβ-fibre responses (*F*_(3,25)_ = 1.466, *P* = 0.2477) (Fig. 1B). We also analysed the effects of Tx3-3 on wind-up of neurones, as a measure of hypersensitivity. Tx3-3 inhibited the wind-up (increases in number of spikes evoked by increasing stimuli number) in both naive (2-way ANOVA, *F*_(3,240)_ = 22.06, *P* < 0.0001) and SNL rats (2-way ANOVA, *F*_(3,240)_ = 28.47, *P* < 0.0001), but SNL rats were more susceptible than naive rats to the dose-dependent inhibition by Tx3-3 (Fig. 2A, B), which was particularly evident by the flattening of the curve of spikes/stimulus x electrical pulses induced by application of 30 pmol/site of Tx3-3 (Fig. 2B). Tx3-3 (30 pmol/site) also inhibited input (number of spikes evoked by the first stimulus of the electrical train) in SNL rats (50.5 ± 14.1% of inhibition; Paired *t* test, *P* = 0.0064), but no difference was found in input of naive rats treated with Tx3-3, at any dose tested (Fig. 2Aa, Bb).

### 3.2. Tx3-3 exhibits higher potency to inhibit naturally evoked neuronal response in spinal nerve ligated than naive rats

**Figure 3.**
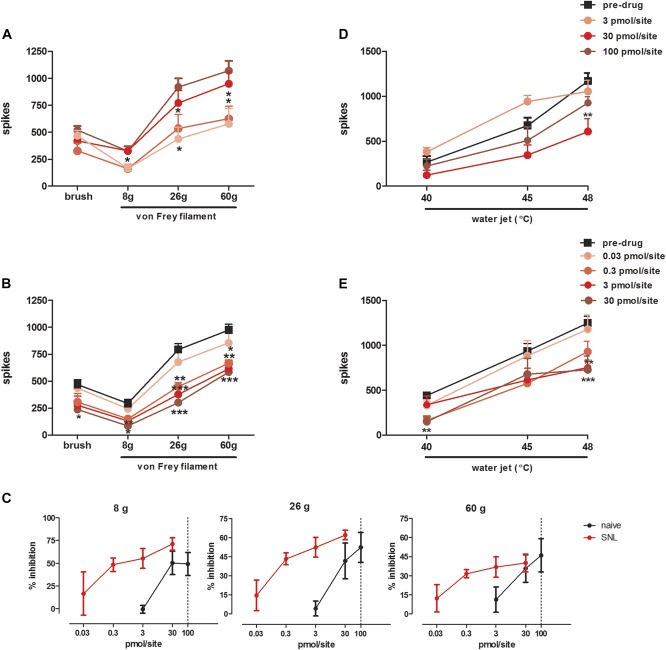
Dose–response curve of Tx3-3 on naturally evoked neuronal responses recorded from naive (A, D) and spinal nerve ligation (SNL) (B, E) rats. Evoked dorsal horn neuronal response to mechanical (A, B) and thermal (D, E) stimuli and dose–response curve of Tx3-3 in naive and SNL rats (C) are shown. Data are expressed as mean maximum inhibition response during the first hour after drug application onto spinal cord of rats (50 µL/site) ± SEM. In SNL rats the neuronal response was evaluated 14 to 17 days after surgery. Each point represents the mean response of 5 to 7 neurones. Vertical lines show the SEM. Statistical analysis was determined using 1-way analysis of variance followed by Dunnett's post hoc test for each mechanical and thermal stimulus. Asterisks denote the significance levels in comparison to predrug values, *P < 0.05, **P < 0.01, ***P < 0.001.

When tested on a range of mechanical stimuli, Tx3-3 exhibited higher potency to inhibit dorsal horn neuronal responses in SNL than naive rats. Tx3-3 inhibited to the same extent neuronal responses evoked by noxious punctate mechanical stimulation (von Frey 26 and 60 g) in SNL and naive rats (Fig. 3A, B), but the same level of inhibition was achieved by a 100-fold lower dose of Tx3-3 in SNL rats (inhibition of 46.7 ± 10.8% was achieved by Tx3-3 at 0.3 pmol/site; *F*_(4,33)_ = 9.781, *P* < 0.0001) in comparison to naive rats (42.2 ± 3.9% was achieved by Tx3-3 at 30 pmol/site; *F*_(3,26)_ = 4.547, *P* = 0.0109) (Fig. 3C). A leftward shift in the dose–response curve of Tx3-3 in SNL rats in comparison to naive rats was also seen in neuronal response evoked by nonnoxious mechanical stimuli (von Frey 8 g). In SNL rats, a significant inhibitory effect on neuronal response evoked by nonnoxious punctate mechanical stimulus (von Frey 8 g) was reached by application of 0.3 pmol/site of Tx3-3 (inhibition of 54.5 ± 8.6%; *F*_(4,33)_ = 4.294, *P* = 0.0066), while 30 pmol/site of Tx3-3 was needed to inhibit neuronal response in naive rats (inhibition of 41.7 ± 13.7%; *F*_(3,26)_ = 3.493, *P* = 0.0297) (Fig. 3C). Moreover, Tx3-3 exhibited inhibitory effect on nonnoxious dynamic mechanical stimulation (brush) in SNL rats (inhibition of 52.2 ± 8.7; *F*_(4,33)_ = 2.840, *P* = 0.0404), but not in naive rats (*F*_(3,26)_ = 2.739, *P* = 0.0637) (Fig. 3A, B).

Neuronal responses to thermal stimulation (40, 45, and 48°C) were also inhibited by spinal application of Tx3-3 in naive and SNL rats. Tx3-3 inhibited neuronal responses evoked by noxious thermal stimulus (48°C) in both naive (inhibition of 41 ± 15%; *F*_(3,23)_ = 3.324, *P* = 0.0375) and SNL rats (inhibition of 38.7% ± 7.6; *F*_(3,25)_ = 6.912, *P* = 0.00015) (Fig. 3D, E). In SNL rats, Tx3-3 mediated a broader inhibition of neuronal response to thermal stimulus, inhibiting also the responses evoked by 40°C (inhibition of 62.5% ± 11.6; *F*_(3,25)_ = 4.532; *P* = 0.0118). Moreover, lower doses of Tx3-3 were required to achieve significant inhibition in SNL rats than in naive rats (Fig. 3D, E).

Overall, the maximum inhibitions produced by Tx3-3 were established around 10 to 20 minutes, and the inhibitory effect lasted approximately 60 minutes (Appendix). No difference was found in timecourse effect of Tx3-3 between naive and SNL rats (data not shown).

## 4. Discussion

In this study, we addressed the effect of the peptide toxin Tx3-3, a P/Q- and R-type VGCC blocker, in in vivo electrophysiological measurements of dorsal horn neuronal responses under physiological and neuropathic conditions. We showed that Tx3-3 mediated differential inhibitory effect on deep dorsal horn neuronal responses under physiological and neuropathic conditions, exhibiting greater potency after nerve injury.

When tested on neuronal responses evoked by electrical stimulation, Tx3-3 exhibited a greater inhibitory profile in SNL than in naive rats. Under neuropathic conditions, Tx3-3 inhibited Aδ, C-fibre, and input responses as well as postdischarge response, while only postdischarge was inhibited in normal animals. Moreover, Tx3-3 produced a greater inhibition of neuronal wind-up in SNL than naive rats. The VGCCs targeted by Tx3-3 are differentially expressed in dorsal horn spinal neurones. P/Q-type VGCCs are expressed on nerve terminals of nonpeptidergic IB4-positive C-fibres, while R-type VGCCs are predominantly localized to somatodendritic compartments^51^ but are also expressed on nerve terminals of peptidergic C-fibres and Aδ-fibres.^17^ Subcellular localization functionally link VGCC subtypes with specific neuronal processes. P/Q-type VGCCs are involved in initiating the release of neurotransmitters at presynaptic sites,^7,9^ while R-type VGCCs play minor role in basal neurotransmitter release, being involved in presynaptic mechanisms of plasticity.^5,14,53^ The inhibition of synaptic transmission through inhibition of neurotransmitter release may account for the effect of Tx3-3 on postdischarge and wind-up responses evoked by electrical stimulation in physiological conditions. In agreement, the blockade of spinal P/Q-type VGCCs by application of ω-agatoxin-IVA in rats attenuated both postdischarge and wind-up neuronal responses.^30^ However, the inhibitory profile of ω-agatoxin-IVA remained unchanged after SNL.^30^ Keeping this in mind, the differential effect mediated by Tx3-3 in neuropathic conditions is thus unlikely to be mediated by its action on P/Q-type VGCCs. Furthermore, classically, P/Q-type VGCC blockers present modest effects against neuropathic pain.^10,50,55^ Interestingly, the inhibitory profile of Tx3-3 on electrically evoked neuronal responses resemble those elicited by the R-type VGCC blocker SNX-482.^29^ Both Tx3-3 and SNX-482, a peptide isolated from the venom of the spider *Hysterocrates gigas*,^34^ inhibited postdischarge and wind-up responses under control conditions, but exhibited a greater inhibitory profile in neuropathic conditions induced by SNL, inhibiting also nociceptive C-fibre and Aδ-fibre responses.^29^ Moreover, like SNX-482, the input and wind-up responses were rather affected by Tx3-3 after neuropathy.

Thus the effect of Tx3-3 on electrically evoked dorsal horn neuronal responses in neuropathic conditions are consistent with a major blockade of R-type VGCCs. In the SNL animals, the wind-up profile differed from that in the control group as the initial response, an index of presynaptic mechanisms delivering the input onto the neurones, was markedly increased, perhaps indicative of enhanced presynaptic spinal mechanisms in keeping with the augmented effects of the toxin after neuropathy.

The antinociceptive properties of Tx3-3 were previously shown in neuropathic pain models of sciatic nerve ligation and diabetic neuropathy.^13^ In the present study, we have addressed the effect of Tx3-3 in in vivo electrophysiology recordings of dorsal horn neurones after the SNL model of chronic neuropathic pain.^23^ In line to the earlier behavioural study, in which Tx3-3 attenuated the tactile allodynia induced by traumatic neuropathy,^13^ here we show that Tx3-3 inhibits dorsal horn neuronal response evoked by innocuous mechanical stimulation in SNL rats. Despite the effect on innocuous mechanical response, Tx3-3 did not affect Aβ-fibres responses. However, electrically evoked responses are suprathreshold and syncronious; by contrast, natural stimuli evoked by 8 g von Frey stimulation applied over 10 seconds are more amenable to inhibition and are likely to activate also Aδ-fibres, which could explain this effect. Importantly, we show that mechanically evoked dorsal horn responses were more sensitive to Tx3-3 in conditions of neuropathy. The higher potency of Tx3-3 in SNL rats suggests a higher affinity of Tx3-3 to VGCC after neuropathy. It has been shown that in neuropathic states the high frequency of fiber activation produces a dynamic change in the VGCC conformational state, switching between resting, activated, and inactivated state.^1^ The access to the inactivated channel is greater during high-frequency neuronal firing conditions, improving the affinity of certain VGCC blockers, as occurring with some ω-conotoxins that exhibit higher potency when binding to the inactivated state of VGCC.^3,47,52,58^ Such state-dependent blockade property could account for the more potent blockade of Tx3-3 during the higher frequency of spontaneous neuronal firing present after SNL.^11^ The higher magnitude of inhibition and dose-related effect of Tx3-3 on wind-up responses in neuropathic rats further support the notion that the reorganization of VGCCs kinetics properties following nerve damage favors the Tx3-3 binding. In this regard, it is noteworthy that neuronal responses evoked by nonnoxious thermal and mechanical stimuli were sensitive to Tx3-3 inhibition preferentially in neuropathic conditions, indicating an enhanced excitability of spinal transmission from afferents that expressed Tx3-3-sensitive VGCCs after neuropathy. In agreement, dorsal horn neuronal response evoked by nonnoxious punctate mechanical stimulus is more sensitive to the R-type VGCC blocker SNX-482 in neuropathic conditions.^29^ Furthermore, P/Q-type VGCCs are also found in the deeper laminae of the dorsal horn,^51^ suggesting its presence on touch-sensitive large fibres, and there are reports of increased levels of P/Q-type in DRG, especially in medium and large myelinated afferent fibers, in neuropathic pain models.^49,56^ Thus, nerve injury may establish a functional reorganization of neuronal phenotypes within the spinal cord, by changing the pattern of expression or activity of some ion channels, including the VGCCs target by Tx3-3, which explains the outcome effect of Tx3-3 on neuronal response evoked by nonnoxious stimuli in neuropathic rats.

It is also worth mentioning the possible role of the auxiliary α2δ subunit of VGCCs in our current results. The α2δ subunits of VGCCs (comprises α2δ-1, α2δ-2, α2δ-3, and α2δ-4) regulate VGCC biophysical properties, trafficking, and membrane expression^2,15,22^ and are upregulated in the dorsal horn spinal cord, mediating spinal hyperexcitability, under neuropathic conditions.^4,25,26^ Recently it was shown that the disruption of the α2δ1 gene expression leads to a delay in development of mechanical hypersensitivity in neuropathic mice, which correlates with a reduction in the response of deep dorsal horn neurones to a range of mechanical stimuli.^37^ Which subtype of Cav2 family is mainly affected by α2δ regulation in neuropathic conditions remains to be clearly defined. However, most aspects of the R-type pore-forming α1 subunit (alpha 1E) are affected by α2δ regulation, including the kinetics of activation-inactivation-deactivation of R-type VGCCs,^40^ and the trafficking of Cav2.1 (which conducts P/Q-type currents) to cell surface is prevented by mutation in the α2δ2 subunits.^20,21^ Therefore, it is possible that the adaptations on VGCC function and expression carried out by α2δ subunits in neuropathic conditions underlie the gain of effect of Tx3-3.

The Tx3-3 affected the neuronal responses evoked by thermal stimulation in naive and SNL rats. These results are in accordance with the previously reported inhibitory effect of Tx3-3 in heat-induced nociceptive pain.^13^ Notably, the doses of Tx3-3 used here are comparable to those used in the above-mentioned behavioral study. In the present study, however, the lower dose of Tx3-3 showed a tendency to increase the thermally evoked neuronal response in naive rats. This may result from the blockade of P/Q-type VGCCs presented on inhibitory interneurones.^48,51^ Likewise, spinal application of the P/Q-type VGCC blocker ω-agatoxin IVA tended to facilitate neuronal responses at lower doses.^30^ However, this phenomenon was not seen in SNL rats, possibly because in this neuropathic condition, R-type VGCCs come into play, as demonstrated by the increased sensitivity of thermally evoked neuronal response to the R-type VGCC blocker SNX-482 in SNL rats.^29^ Therefore, possibly, the neuropathy may cause a pathophysiological upregulation of R-type VGCC activity (or promote a given conformational state) that favors the action of Tx3-3 on it, explained by the enhanced sensitivity of SNL rats to Tx3-3 inhibitory effect on thermally evoked neuronal responses (as lower doses were needed to achieve inhibitory effect in SNL rats compared to control rats).

This is the first electrophysiological study addressing the effects of Tx3-3 on sensory transmission in spinal cord of rats. The present data extend results from previous behavioural studies, showing a prevalent antinociceptive effect of Tx3-3 on neuropathic states and suggest the R-type VGCC as the main target of such action.

## 5. Concluding remarks

A significant functional role of VGCCs in neuropathic pain mechanisms has been substantiated by studies using toxins isolated from animal venoms.^38,54^ Here, we showed that the toxin Tx3-3, a P/Q- and R-type VGCC blocker isolated from the venom of the spider *P. nigriventer,* mediated differential inhibitory effect on deep dorsal horn neuronal responses under physiological and neuropathic pain conditions, exhibiting greater potency after nerve damage. The profile of action of Tx3-3 highlights the role of VGCCs, drawing attention to R-type VGCC, in pathological pain and may provide insight into the development of specific analgesics for the treatment of neuropathic pain.

## Disclosures

The authors have no conflict of interest to declare.

Supported by the Wellcome Trust London Pain Consortium strategic award. G. D. Dalmolin is supported by Capes/Toxinologia (process 8849-11-0). There are not any financial or other relationships that might lead to a conflict of interest in this study.
